# Results of surgical management in mechanical mitral valve thrombosis

**DOI:** 10.1186/1749-8090-10-S1-A188

**Published:** 2015-12-16

**Authors:** Salim Chibane, Abdelmalek Bouzid, Mohamed Atbi, Boukri Hamouda, Youcef Larabi, Tarik Hamdi, Reda DjilaliSayeh, Halima Larbi, Ramdane Amar Ould Abderahmane

**Affiliations:** 1 Department of Cardiac Surgery, 1st November Hospital, Oran 31000, Algéria

## Background/Introduction

Valve thrombosis is a serious complication of heart valve surgery. It most often involves mechanical valve in mitral position and may be life-threatening in the short term period, necessitating prompt treatment. Among therapeutic options, surgery leads to complete success of deobstruction but with a high morbi-mortality rate.

## Aims/Objectives

The purpose of this study is to review the results of our experience in surgical management of mechanical mitral valve thrombosis.

## Method

This is a retrospective study including fifteen patients operated on in our institution for mitral valve thrombosis between 2012 through 2014. Ten patients (66.7%) were operated on as emergent cases (within 24 h after admission) and five patients (33.3%) were elective (3-5 days after admission). Mean age was 45 years and 12 were females. All patients had a mechanical valve implanted in the mitral position; mean time between first and second intervention was 3.9 years [7 day-19 years]. Clinically 14 patients (93.3%) were in NYHA stage III-IV on admission, seven had pulmonary edema (46.7%) and 6 had hemodynamic instability (40%) necessitating inotropic support. Mean preoperative mitral gradient was 26.4 mmHg. All patients underwent a surgical procedure, consisting of valve replacement in 14 patients and thrombectomy in one case. Five patients had associated tricuspid annuloplasty.

## Results

Six patients died postoperatively which represents a 40% mortality rate. This occurred mainly in patients with hemodynamic instability and NYHA class IV. Good results were achieved in patients with stable clinical status (NYHA class II-III) with no mortality in this group. All other patients were discharged after an uneventful postoperative course and no patients had recurrence of valve thrombosis at a mean follow up of 17 months.

## Discussion/Conclusion

MVT is a serious complication with a dark spontaneous prognosis. It management should be as short as possible between diagnosis and intervention. In stable cases, surgery leads to good results with low morbi-mortality rates. In case of hemodynamic instability, other alternatives should be considered, including thrombolysis because operative morbidity and mortality remain high in these cases.

